# Estimation of inter-laboratory reference change values from external quality assessment data

**DOI:** 10.11613/BM.2021.030902

**Published:** 2021-08-05

**Authors:** Michael Paal, Katharina Habler, Michael Vogeser

**Affiliations:** 1 Institute of Laboratory Medicine, University Hospital, LMU Munich, Germany

**Keywords:** reference change values, inter-laboratory, external quality assessment, measurement uncertainty, biological variation

## Abstract

**Introduction:**

It is common for patients to switch between several healthcare providers. In this context, the long-term follow-up of medical conditions based on laboratory test results obtained from different laboratories is a challenge. The measurement uncertainty in an inter-laboratory context should also be considered in data mining research based on routine results from randomly selected laboratories. As a proof-of-concept study, we aimed at estimating the inter-laboratory reference change value (IL-RCV) for exemplary analytes from publicly available data on external quality assessment (EQA) and biological variation.

**Materials and methods:**

External quality assessment data of the Reference Institute for Bioanalytics (RfB, Bonn, Germany) for serum creatinine, calcium, aldosterone, PSA, and of whole blood HbA1c from campaigns sent out in 2019 were analysed. The median CVs of all EQA participants were calculated based on 8 samples from 4 EQA campaigns *per* analyte. Using intra-individual biological variation data from the EFLM database, positive and negative IL-RCV were estimated with a formula based on log transformation under the assumption that the analytes under examination have a skewed distribution.

**Results:**

We estimated IL-RCVs for all exemplary analytes, ranging from 13.3% to 203% for the positive IL-RCV and - 11.8% to - 67.0% for the negative IL-RCV (serum calcium - serum aldosterone), respectively.

**Conclusion:**

External quality assessment data together with data on the biological variation – both freely available – allow the estimation of inter-laboratory RCVs. These differ substantially between different analytes and can help to assess the boundaries of interoperability in laboratory medicine.

## Introduction

A major objective of laboratory medicine is standardization, which is intended to enable the interoperability of results from different test sites (*1*, *2*). This is essential for both the development and application of clinical algorithms with decision limits based on laboratory values and for the long-term follow-up of patients with chronic diseases. Similarly, interoperability of laboratory results is a major challenge for scientific investigations that rely on unselected and not individually traceable routine data (*e.g.*, big data approach). Especially the implementation of universal standards for identifying medical laboratory observations in electronic records, such as the Logical Observation Identifiers Names and Codes (LOINC) code system, has fuelled the mining of lab data (*3*).

Discrepancies in values due to insufficient standardization can in principle be compensated by the determination of method-specific reference values; for scientific applications, the evaluation can then, for example, be carried out as x-fold of a certain reference range value. However, this is currently not practiced for essential laboratory analytes. Indeed, clinical guidelines addressing analytes such as prostate-specific antigen (PSA) or creatinine do not consider possible methodological discrepancies at all (*4*, *5*).

So far, there is no comprehensive data available to assess the limits of interoperability of standard laboratory analytes. Results of external quality assessment (EQA) schemes, some of which are publicly available, are an attractive data source in this context.

Reference change values (RCV) or critical differences have become established for the estimation of a true intra-individual dynamic in the follow-up of a patient beyond the measurement uncertainty (*6*). The calculation of the RCV takes into account the analytical variation of the measurement of an analyte (CV_A_) and the intra-individual biological variation (CV_I_) of the respective analyte (*7*). The RCV indicates the percentage by which two sequential results of a patient must differ if an actual biological change in the concentration must be assumed with a high degree of probability. The estimation of RCVs in the current application assumes that follow-up measurements are done in one and the same laboratory under unchanged conditions.

**Figure 1:**
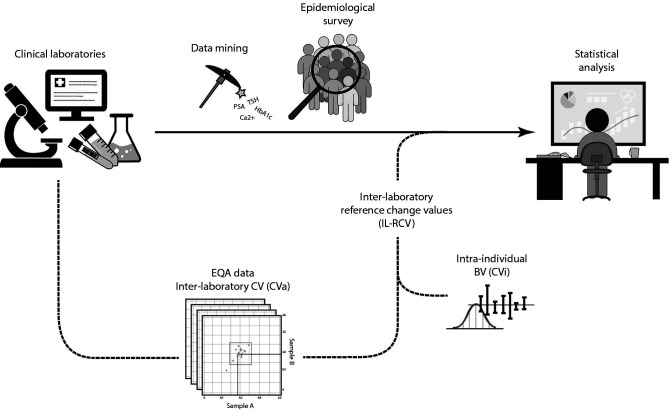
Scheme of calculation of inter-laboratory reference change values. EQA analytical variation from participating clinical laboratories and intra-individual biological variation (CV_I_) data can be used to calculate analyte-specific IL-RCV, which may be used for a comprehensive evaluation of combined inter-laboratory data sets from unselected laboratories with heterogenous measurement procedures, *e.g.*, as typically extracted in data mining and epidemiological studies. IL-RCV – inter-laboratory reference change values. EQA – external quality assessment. CV_A_ – analytical variation. CV_I_ – intra-individual biological variations.

The evaluations of EQA campaigns across all participants – without method-specific evaluations – can be used to estimate CV_A_ in an inter-laboratory setting. The database of the European Federation of Laboratory Medicine (EFLM) provides a good basis for the CV_I_ to be assumed (*8*). Calculated inter-laboratory RCV (IL-RCV) could in turn be used for a critical appraisal of inter-laboratory records as exploited in epidemiological and data mining studies (Figure 1).

The aim of the study was to provide a proof-of-concept for the feasibility of estimating an IL-RCV from the above-mentioned data sources as a basis for assessing the interoperability of a particular analyte and to reflect the limitations of this approach. We addressed five representative standard analytes from different biochemical classes.

## Materials and methods

### Materials

External quality assessment data of the Reference Institute for Bioanalytics (Rfb, Bonn, Germany) were obtained from the freely accessible web resources (*9*). Four EQA programs were distributed in four campaigns in 2019 (identification: KS 1-4 2019; HM 1-4 2019; TM 1-4 2019; GH 1-4 2019). The proficiency test samples are lyophilized materials. Data sets were evaluated for the five analytes: serum calcium, creatinine, aldosterone, PSA, and whole blood haemoglobin A1c (HbA1c). For each EQA sample, the mean value of the concentrations found by the participants, the CV observed for each analyte and sample from the participants’ data (as total analytical CV, CV_A_), the number of participants in the respective campaign, and the number of different methods stated in the EQAs report were assessed.

The data on biological variation (within-subject BV, CV_I_) of the five exemplary analytes were taken from the respective database of the EFLM and, in the case of serum calcium, from a printed publication (*8*, *10*).

### Methods

There are several ways to calculate the RCV (*7*). The traditional approach introduced by Harris and Yasaka is RCV = 2^1/2^ x Z_α_ x (CV_A_^2^ + CV_I_^2^)^1/2^ (*11*). This is used to calculate symmetrical limits of the RCV for analytes that follow a normal distribution, where Z_α_ is defining the number of standard deviations appropriate for the probability.

Given that many laboratory analytes have skewed rather than normal distributions, the log-normal approach is typically considered the best approach to determining RCV values (*12*, *13*). Accordingly, we applied the log-normal approach, described by Fokkema *et al.* to calculate asymmetrical limits for the positive (upward) and negative (downward) reference change values (RCV_pos_, RCV_neg_) with the following equation RCV_pos/neg_ = 100% x (exp (± Z_α_ x 2^1/2^ (SD_A_^2^ + SD_I_^2^)^1/2^ - 1) (*14*). The calculation of SD_A_^2^ is performed as SD_A_^2^ = ln (CV_A_^2^+1), while SD_I_^2^ is calculated as SD_I_^2^ = ln (CV_I_^2^+1).

In many clinical situations, decision-making is typically based on the assessment of a significant rise or fall of a target analyte. We, therefore, set Z_α_ to 1.96, leading to 95% probability (P < 0.05) that is regarded as significant.

## Results

For all five exemplary analytes, the estimation of an IL-RCV was found possible. The results are summarized in Table 1. The range of IL-RCVs of the analytes investigated was considerable and RCV_pos_ ranged from 13.3% to 203% and RCV_neg_ from - 11.8% to - 67.0% for serum calcium and aldosterone, respectively.

**Table 1:** Estimation of inter-laboratory positive and negative reference change values for five exemplary analytes based on data from external quality assessment schemes

**Analyte**	**Unit**	Lowest – highest concentration (mean)*	**Median of results^†^**	**CV_A_ (%)^‡^**	**CV_I_ (%)^§^**	**Estimated** **RCV_pos_/_neg_** **(%)^║^**	**Number of methods^¶^**	Number of EQA participants**
Creatinine	µmol/L	115 – 478	168	4.7	4.5	+19.7 / - 16.5	14	645
Calcium	mmol/L	1.81 – 3.35	2.20	3.1	3.3	+13.3 / - 11.8	12	615
Aldosterone	nmol/L	0.3 – 20.1	0.8	18.6	36.6	+203 / - 67.0	8	203
PSA	µg/L	0 – 14.0	0.4	18.8	6.8	+73.1 / - 42.2	13	804
HbA1c	mmol/mol	36.0 – 71.2	51.5	5.1	1.7	+16.1 / - 13.9	12	810
*Lowest/highest mean concentration observed in the four EQA campaigns and each two samples *per* analyte. ^†^Median of 8 means observed in the four EQA campaigns and each two samples *per* analyte. ^‡^Median CV of all participants observed in the four EQA campaigns studied, each two samples (median of 8 CVs). ^§^Biological variation according to EFLM data base and for serum calcium (8,10). ^║^RCV according to Fokkema *et al.* (14). ^¶^Mean number of methods in the four campaigns studied. **Mean number of participants *per* EQA scheme from four campaigns in 2019. PSA – prostate-specific antigen. HbA1c – haemoglobin A1c. CV – coefficient of variation. CV_A_ – analytical variation of all participants. CV_I_ – intra-individual biological variations. RCV_pos/neg_ – positive (upward) and negative (downward) reference change values. EQA – external quality assessment. EFLM – European Federation of Clinical Chemistry and Laboratory Medicine.								

## Discussion

We have shown that the estimation of IL-RCVs from publicly available EQA and intra-individual biological variation data is possible for essential laboratory analytes.

We observed very large differences for RCV_pos_ and RCV_neg_ between five exemplary analytes from different biochemical classes applying the formula for log-normal RCV calculation (*14*). While IL-RCV for serum calcium, creatinine, and whole blood HbA1c were found below 20%, the IL-RCV for serum aldosterone must be assumed to be well above 150% for positive and 50% for negative changes. This means that if patient’s samples are sent to randomly selected laboratories for follow-up testing, a real change in the concentration of the analyte in the biological system can only be assumed with a high degree of probability (95%) if the rise in values exceeds 203% in case of serum aldosterone. An increase of reported concentrations below cannot be considered a real change within the limits of measurement uncertainty in a between-laboratory setting.

We recognize that our approach has some limitations. External quality assessment samples are typically processed specimens (*e.g.*, for virus inactivation), which can lead to limitations regarding commutability. Especially lyophilized control materials can show commutability problems and behave differently than patient samples. Accordingly, analytical variation values calculated from lyophilized materials may not correspond to the analytical variation calculated on the basis of commutable material. It is therefore possible that the real IL-RCV is lower than we observed when using exclusively unprocessed single-donor material in EQA. Corresponding data should in principle be generated but are currently not publicly available.

The Biological Variation Data Critical Appraisal Checklist (BIVAC) regularly updates the biological variation data from the EFLM database from systematic reviews and published studies. Still, it must be taken into account that the determination of biological variation data is not always based on very large data sets and that it has uncertainties, given that published CV_I_ may not necessarily match the investigated patient cohort (*15*).

Our procedure can be used to assess the measurement uncertainty in epidemiological surveys and data mining studies, such as the Medical Informatics Initiative Germany, when data from unselected laboratories are used (*16*). They allow an estimation of the limits of interoperability of routine data collected with today’s heterogeneous standard procedures and kits and should be considered when interpreting corresponding combined data sets. Furthermore, IL-RCV may assist in the interpretation of changes in patient serial results obtained from different laboratories. For a more comprehensive assessment, multiple EQA schemes and accessions might be combined to establish a corresponding expanded data set for inter-laboratory analytical variation.

A notable caveat in large-scale data mining studies is the usage of pooled data from multiple laboratories. It must be emphasized that corresponding IL-RCVs are not exact mathematical calculations that can be transferred to individual evaluations without exception but are rather estimates that may be useful for critical appraisal, in particular of data mining studies.

We conclude that EQA data together with data on the biological variation – both freely available – allow the estimation of inter-laboratory RCVs. These differ substantially between different analytes and can help to assess the boundaries of interoperability in laboratory medicine.
